# SARS-CoV-2 infection hospitalization, severity, criticality, and fatality rates in Qatar

**DOI:** 10.1038/s41598-021-97606-8

**Published:** 2021-09-14

**Authors:** Shaheen Seedat, Hiam Chemaitelly, Houssein H. Ayoub, Monia Makhoul, Ghina R. Mumtaz, Zaina Al Kanaani, Abdullatif Al Khal, Einas Al Kuwari, Adeel A. Butt, Peter Coyle, Andrew Jeremijenko, Anvar Hassan Kaleeckal, Ali Nizar Latif, Riyazuddin Mohammad Shaik, Hadi M. Yassine, Mohamed G. Al Kuwari, Hamad Eid Al Romaihi, Mohamed H. Al-Thani, Roberto Bertollini, Laith J. Abu-Raddad

**Affiliations:** 1 Infectious Disease Epidemiology Group, Weill Cornell Medicine-Qatar, Cornell University, Doha, Qatar; 2 World Health Organization Collaborating Centre for Disease Epidemiology Analytics On HIV/AIDS, Sexually Transmitted Infections, and Viral Hepatitis, Weill Cornell Medicine–Qatar, Cornell University, Qatar Foundation – Education City, P.O. Box 24144, Doha, Qatar; 3 Department of Population Health Sciences, Weill Cornell Medicine, Cornell University, New York, NY USA; 4 Department of Mathematics, Statistics, and Physics, Qatar University, Doha, Qatar; 5 Department of Epidemiology and Population Health, American University of Beirut, Beirut, Lebanon; 6 Hamad Medical Corporation, Doha, Qatar; 7 Biomedical Research Center, Qatar University, Doha, Qatar; 8 Department of Biomedical Science, College of Health Sciences, Member of QU Health, Qatar University, Doha, Qatar; 9 Primary Health Care Corporation, Doha, Qatar; 10 Ministry of Public Health, Doha, Qatar

**Keywords:** Viral infection, Respiratory signs and symptoms, Respiratory tract diseases

## Abstract

The SARS-CoV-2 pandemic resulted in considerable morbidity and mortality as well as severe economic and societal disruptions. Despite scientific progress, *true* infection severity, factoring both diagnosed and undiagnosed infections, remains poorly understood. This study aimed to estimate SARS-CoV-2 age-stratified and overall morbidity and mortality rates based on analysis of extensive epidemiological data for the pervasive epidemic in Qatar, a country where < 9% of the population are ≥ 50 years. We show that SARS-CoV-2 severity and fatality demonstrate a striking age dependence with low values for those aged < 50 years, but rapidly growing rates for those ≥ 50 years. Age dependence was particularly pronounced for infection criticality rate and infection fatality rate. With Qatar’s young population, overall SARS-CoV-2 severity and fatality were not high with < 4 infections in every 1000 being severe or critical and < 2 in every 10,000 being fatal. Only 13 infections in every 1000 received any hospitalization in acute-care-unit beds and < 2 in every 1000 were hospitalized in intensive-care-unit beds. However, we show that these rates would have been much higher if Qatar’s population had the demographic structure of Europe or the United States. Epidemic expansion in nations with young populations may lead to considerably lower disease burden than currently believed.

## Introduction

The pandemic caused by the severe acute respiratory syndrome coronavirus 2 (SARS-CoV-2) infection and associated Coronavirus Disease 2019 (COVID-19) continue to be a global health challenge^1^. Aside from direct implications on morbidity and mortality^1^, the pandemic led to severe economic and societal disruptions^2^, a consequence of the social and physical distancing restrictions imposed to slow SARS-CoV-2 transmission in view of its severity and fatality.

One of the most affected countries by this pandemic is Qatar, a peninsula in the Arabian Gulf region with a population of 2.8 million^3,4^. With its unique demographic and residential dwelling structure where 60% of the population are expatriate craft and manual workers (CMWs) living in large shared housing accommodations^3,4^, Qatar experienced a pervasive epidemic with > 60,000 laboratory-confirmed infections per million population as of November 23, 2020^5,6^. The epidemic grew rapidly starting from March 2020, peaked in late May 2020, then rapidly declined in subsequent weeks, and had been in a stable low-incidence phase up to end of 2020 (Supplementary Figure S1). A series of serological studies completed by November 23, 2020 suggested that about half of the population have already been infected^6–9^.

With a well-resourced public healthcare structure and a centralized and standardized data-capture system for all SARS-CoV-2 testing and COVID-19 care, Qatar has one of the most extensive databases to characterize this epidemic and its toll^6^. In addition to large-scale polymerase chain reaction (PCR) and serological testing, multiple population-based PCR and serological surveys have been conducted to date^6–9^. As of November 23, 2020, cumulative overall testing rates exceeded 638,000 per million population for PCR and 105,000 per million population for antibodies^10^. A comprehensive clinical characterization has also been completed for the hospitalized COVID-19 cases through individual chart reviews by trained medical personnel, including infection severity classification as per the World Health Organization (WHO) guidelines^11^.

Given the pervasive and advanced nature of the epidemic and availability of extensive epidemiological data, Qatar provides a unique opportunity to assess the extent of SARS-CoV-2 morbidity and fatality. We aimed in this study to estimate the age-stratified and overall infection acute-care and intensive-care-unit (ICU) hospitalization rates, infection severity and criticality rates, and infection fatality rate.

## Methods

### Mathematical model and parameterization

Building on our previously developed SARS-CoV-2 models^12–18^, an age-structured deterministic mathematical model was constructed to describe SARS-CoV-2 transmission dynamics and disease progression in the population from the epidemic onset up to November 22, 2020 (Supplementary Figure S2). Susceptible individuals in each age group were at risk of acquiring the infection based on their infectious contact rate per day, age-specific susceptibility to the infection, and an age-mixing matrix defining mixing between individuals in the different age groups. Following a latency period, infected individuals developed an infection that either did not require hospitalization, or that required hospitalization in an acute-care bed or in an ICU bed. Individuals admitted to an ICU bed had an additional risk for COVID-19 mortality. The model further included compartments tracking infection severity (asymptomatic/moderate/mild infection, severe infection, or critical infection as per WHO severity classification)^11^. Population movement between model compartments was described using a set of coupled nonlinear differential equations (Supplementary Text S1).

The model was parameterized using best available data for SARS-CoV-2 natural history and epidemiology. Model parameters, definitions, and justifications can be found in Supplementary Tables S1 and S2. The size and demographic structure of the population of Qatar were based on a population census conducted by Qatar’s Planning and Statistics Authority^3^.

### Model fitting and analyses

The model was fitted to extensive time-series and age-stratified data for PCR laboratory-confirmed infections, PCR testing positivity rate, antibody testing positivity rate, PCR and serological surveys, daily hospital admissions in acute-care and ICU beds, hospital occupancy in acute-care and ICU beds, incidence of severe and critical infections as per WHO classification^11^, and COVID-19 deaths (further details in Supplementary Text S2). A Bayesian method, based on the incremental mixture importance sampling with shot-gun optimization^19,20^, was used to fit the model to the different data sources and to estimate the mean and 95% credible interval (CI) for each estimated parameter (Supplementary Text S2), such as the mean duration of acute-care hospitalization and the mean duration of ICU-care hospitalization. The model was coded, fitted, and analyzed using MATLAB R2019a^21^.

### Outcome measures

Table 1 provides a listing of each outcome measure estimated in this study, its definition, and its interpretation. Two sets of outcome measures were generated. The first set includes *crude case rates*, such as the crude case fatality rate, calculated as the cumulative number of a disease outcome (say COVID-19 death) over the cumulative number of *documented* (that is PCR laboratory-confirmed) infections.

**Table 1:** Crude case rates and infection rates estimated in this study.

Outcome measure	Definition	Interpretation
**Crude case rates-data estimation**		
1. Crude acute-care *and* ICU bed hospitalization rate	Cumulative number of hospital admissions into acute-care or ICU beds over the cumulative number of *documented* PCR laboratory-confirmed infections	Proportion of PCR laboratory-confirmed infections that progressed to hospital admission into acute-care or ICU beds
2. Crude case severity *and* criticality rate	Cumulative number of COVID-19 severe or critical infections* over the cumulative number of *documented* PCR laboratory-confirmed infections	Proportion of PCR laboratory-confirmed infections that progressed to become severe or critical
3. Crude case fatality rate	Cumulative number of COVID-19 deaths over the cumulative number of *documented* PCR laboratory-confirmed infections	Proportion of PCR laboratory-confirmed infections that ended in COVID-19 death
**Infection rates-model estimation**		
1. Infection acute-care bed hospitalization rate	Cumulative number of hospital admissions into acute-care beds over the cumulative number of infections, *documented and undocumented*	Proportion of infections that progressed to acute-care bed hospital admission
2. Infection ICU bed hospitalization rate	Cumulative number of hospital admissions into ICU beds over the cumulative number of infections, *documented and undocumented*	Proportion of infections that progressed to ICU bed hospital admission
3. Infection severity rate	Cumulative number of COVID-19 severe infections* over the cumulative number of infections, *documented and undocumented*	Proportion of infections that progressed to become severe infections
4. Infection criticality rate	Cumulative number of COVID-19 critical infections* over the cumulative number of infections, *documented and undocumented*	Proportion of infections that progressed to become critical infections
5. Infection fatality rate	Cumulative number of COVID-19 deaths over the cumulative number of infections, *documented and undocumented*	Proportion of infections that ended in COVID-19 death
**Combined infection rates-model estimation**		
1. Infection acute-care *and* ICU bed hospitalization rate	Cumulative number of admissions into acute-care or ICU beds over the cumulative number of infections, *documented and undocumented*	Proportion of infections that progressed to acute-care or ICU bed hospital admission
2. Infection severity *and* criticality rates	Cumulative number of COVID-19 severe or critical infections* over the cumulative number of infections, *documented and undocumented*	Proportion of infections that progressed to become severe or critical infections

*Per World Health Organization (WHO) infection severity classification^11^.

The second set includes *infection rates*, such as the infection fatality rate, calculated as the cumulative number of a disease outcome (say COVID-19 death) over the model-estimated cumulative number of infections, *documented and undocumented*.

Two separate criteria for classifying morbidity were used: one based on actual recorded hospital admission (acute-care or ICU) and one based on clinical presentation as per WHO classification of disease severity. While the two are overlapping with severe cases typically admitted to acute-care beds, and critically ill cases admitted to ICU beds, a significant fraction of mild or moderately ill cases were hospitalized out of caution. Moreover, hospitalization was used as a form of case isolation earlier in the epidemic. Of note that the health system in Qatar remained well within its threshold even at the epidemic peak towards end of May 2020.

The diagnosis (detection) rate was further calculated as the cumulative number of infections that were documented (that is with PCR laboratory-confirmed diagnosis) over the model-estimated cumulative number of infections, *documented and undocumented*.

### Ethical approvals

This study was approved by the HMC and Weill Cornell Medicine-Qatar Institutional Review Boards.

## Results

**Figure 1:**
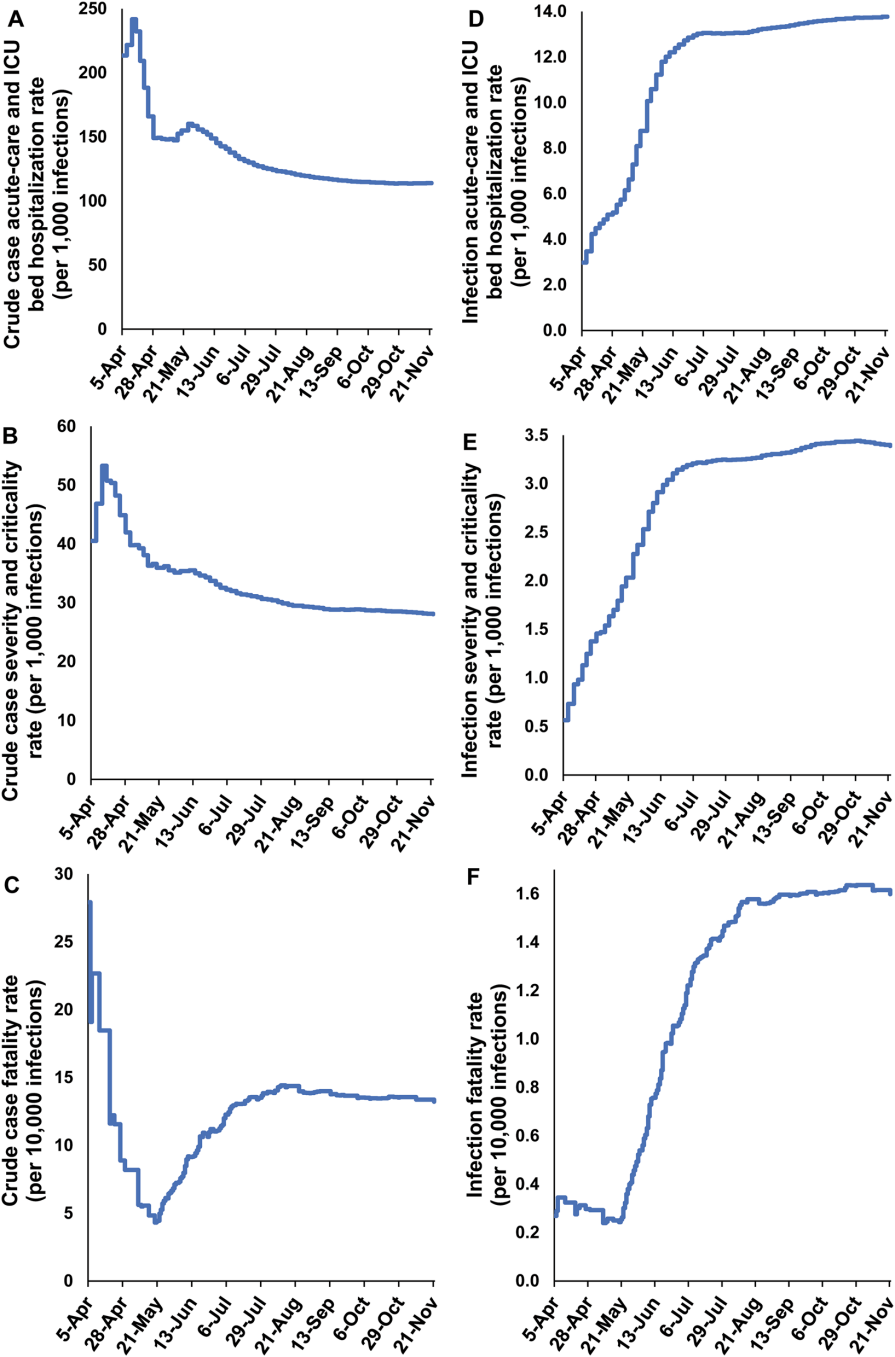
Temporal trend in (**A**) crude case acute-care and ICU bed hospitalization rate, (**B**) crude case severity and criticality rate, (**C**) crude case fatality rate, (**D**) infection acute-care and ICU bed hospitalization rate, (**E**) infection severity and criticality rate, and (**F**) infection fatality rate. Classification of infection severity and criticality was per WHO infection severity classification^11^.

Figure 1A shows the crude case acute-care *and* ICU bed hospitalization rate versus time from the epidemic onset up to November 22, 2020. The rate was rather stable, but with a slightly declining trend, and was assessed at 113.9 acute-care *and/or* ICU hospital admissions per 1000 laboratory-confirmed infections on November 22, 2020. As of this date, a total of 18,509 acute-care and 1759 ICU hospital admissions had been registered.

Figure 1B shows the crude case severity *and* criticality rate versus time. The rate was rather stable, but with a slightly declining trend, and was assessed at 28.0 severe *and/or* critical cases per 1000 laboratory-confirmed infections on November 22, 2020. As of this date, a total of 4127 severe and 863 critical infections had been registered.

Figure 1C shows the crude case fatality rate versus time. The rate increased over time, as expected with the weeks-long delay between infection and eventual COVID-19 death, but then stabilized as the epidemic entered its low but stable infection incidence phase (Supplementary Figure S1). The rate was assessed at 13.2 deaths per 10,000 laboratory-confirmed infections on November 22, 2020. As of this date, a total of 235 COVID-19 deaths had been registered.

The model produced robust fits to each dataset. Supplementary Table S3 summarizes the goodness-of-fit. Supplementary Figures S3–S7 show the *age-specific* posterior distributions of the infection acute-care bed hospitalization rate (Supplementary Figure S3), infection ICU bed hospitalization rate (Supplementary Figure S4), infection severity rate (Supplementary Figure S5), infection criticality rate (Supplementary Figure S6), and infection fatality rate (Supplementary Figure S7). Meanwhile, Supplementary Figures S8–S9 show the *overall* (total population of all age groups) infection acute-care bed hospitalization rate (Supplementary Figure S8A), infection ICU bed hospitalization rate (Supplementary Figure S8B), infection severity rate (Supplementary Figure S8C), infection criticality rate (Supplementary Figure S8D), and infection fatality rate (Supplementary Figure S9).

**Figure 2:**
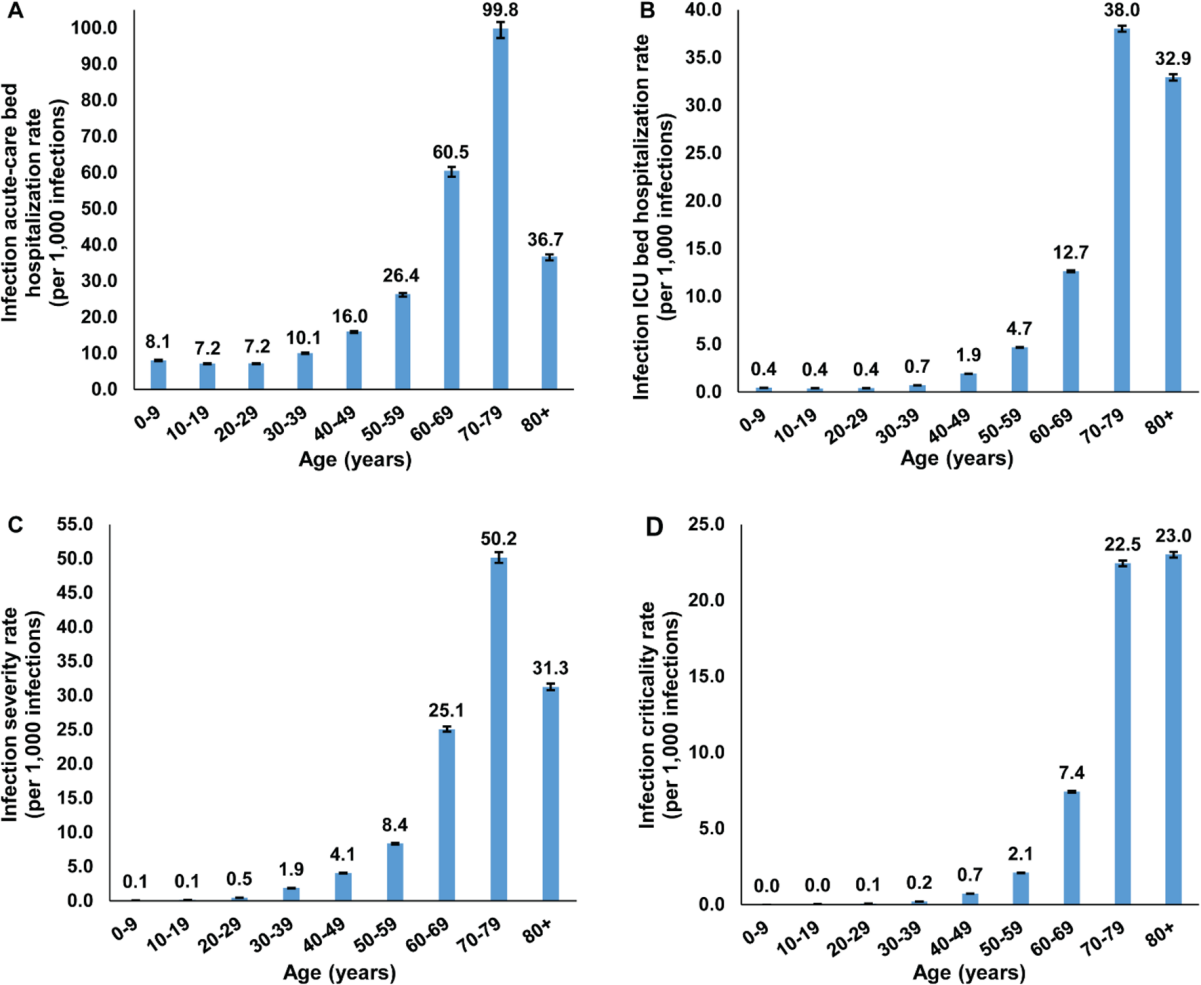
The *age-specific* (**A**) infection acute-care bed hospitalization rate, (**B**) infection ICU bed hospitalization rate, (**C**) infection severity rate, and (**D**) infection criticality rate. Classification of infection severity and criticality was per WHO severity classification^11^.

**Figure 3:**
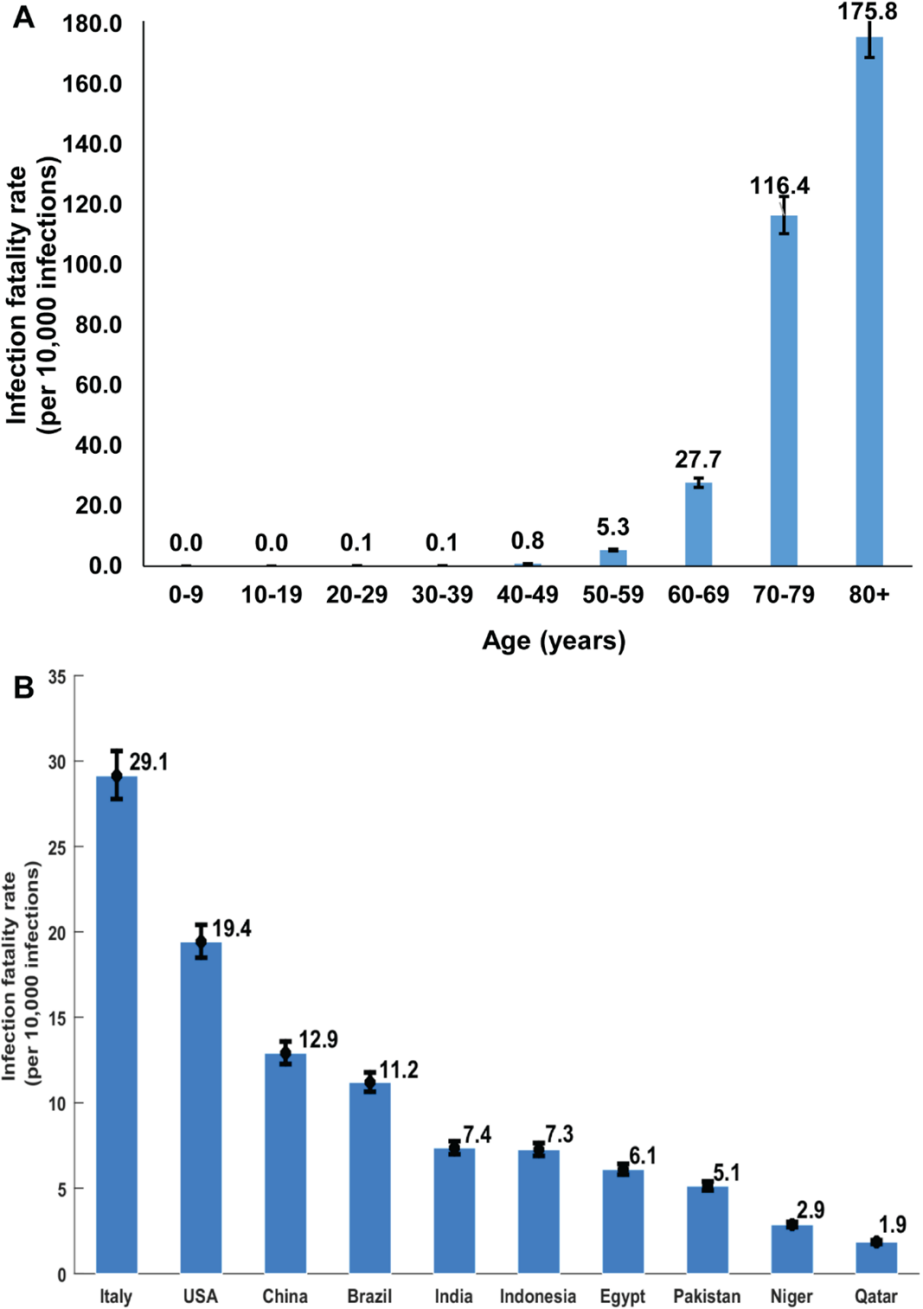
(**A**) The *age-specific* infection fatality rate in Qatar. (**B**) Estimated overall (total population of all age groups) infection fatality rate for several select countries that are characterized by diverse demographic structures. The estimates were generated by applying the Qatar-estimated *age-specific* infection fatality rate to the age structure of the population in each country. Population size and demographic age-structure of each country were extracted from the United Nations World Population Prospects database^22^.

Table 2, Figs. 2, and 3A show the estimated mean and 95% CI of all *age-specific infection rate* measures. All rates showed very strong age dependence. Measures increased steadily with age, with low values for those < 50 years of age, but very rapidly growing rates for those ≥ 50 years of age. The strong age dependence was even more pronounced for infection ICU bed hospitalization rate (Fig. 2B), infection criticality rate (Fig. 2D), and infection fatality rate (Fig. 3A).

**Table 2:** Estimated mean and 95% credible interval (CI) of the age-specific infection acute-care and ICU bed hospitalization rates, infection severity and criticality rates, and infection fatality rate.

Age group (years)	Infection acute-care bed hospitalization rate (per 1000 infections)	Infection ICU bed hospitalization rate (per 1000 infections)	Infection severity rate (per 1000 infections)	Infection criticality rate (per 1000 infections)	Infection fatality rate (per 10,000 infections)
	Mean (95% CI)	Mean (95% CI)	Mean (95% CI)	Mean (95% CI)	Mean (95% CI)
0–9	8.06 (7.85–8.20)	0.44 (0.43–0.46)	0.07 (0.07–0.08)	0.00 (0.00–0.00)	0.00 (0.00–0.00)
10–19	7.15 (6.96–7.27)	0.39 (0.38–0.41)	0.13 (0.13–0.13)	0.04 (0.04–0.04)	0.00 (0.00–0.00)
20–29	7.19 (7.02–7.28)	0.40 (0.39–0.41)	0.47 (0.46–0.48)	0.08 (0.08–0.08)	0.12 (0.11–0.13)
30–39	10.07 (9.88–10.18)	0.71 (0.69–0.72)	1.86 (1.83–1.89)	0.21 (0.21–0.21)	0.09 (0.09–0.10)
40–49	16.00 (15.65–16.17)	1.92 (1.90–1.94)	4.05 (3.99–4.11)	0.73 (0.72–0.73)	0.75 (0.70–0.80)
50–59	26.35 (25.69–26.68)	4.67 (4.63–4.70)	8.35 (8.22–8.48)	2.10 (2.08–2.12)	5.31 (4.99–5.65)
60–69	60.46 (58.82–61.53)	12.65 (12.54–12.75)	25.08 (24.70–25.48)	7.43 (7.37–7.49)	27.68 (26.15–29.26)
70–79	99.84 (97.23–101.65)	38.03 (37.72–38.32)	50.16 (49.39–50.95)	22.45 (22.25–22.62)	116.44 (110.43–122.83)
80 +	36.73 (35.76–37.37)	32.94 (32.60–33.26)	31.26 (30.78–31.76)	23.02 (22.82–23.19)	175.76 (168.92–183.17)
Overall population	13.10 (12.82–13.24)	1.60 (1.58–1.61)	3.06 (3.01–3.10)	0.68 (0.67–0.68)	1.85 (1.74–1.95)

Classification of infection severity and criticality was per WHO infection severity classification^11^.

The *overall* (total population of all age groups) infection acute-care bed hospitalization rate was estimated at 13.10 (95% CI 12.82–13.24) per 1000 infections, infection ICU bed hospitalization rate at 1.60 (95% CI 1.58–1.61) per 1000 infections, infection severity rate at 3.06 (95% CI 3.01–3.10) per 1000 infections, infection criticality rate at 0.68 (95% CI 0.67–0.68) per 1000 infections, and infection fatality rate at 1.85 (95% CI 1.74–1.95) per 10,000 infections.

Applying the above estimated *age-specific* infection fatality rate in Qatar to the age structure of other national populations^22^, the overall infection fatality rate for these countries varied substantially just because of the differences in the demographic structure (Fig. 3B).

Figure 1D shows the infection acute-care *and* ICU bed hospitalization rate versus time. The rate increased over time, as expected with the delay between infection and hospital admission, unlike the corresponding crude case rate which decreased over time as PCR testing was expanded and more and more of the mild and asymptomatic infections were diagnosed. The infection acute-care and ICU bed hospitalization rate then stabilized (Fig. 1D) as the epidemic peaked and started to decline (Supplementary Figure S1). The rate was assessed at 13.8 hospital admissions per 1000 infections on November 22, 2020. A similar pattern was found for the infection severity *and* criticality rate (Fig. 1E) and infection fatality rate (Fig. 1F), which were assessed at 3.4 cases per 1000 infections and 1.6 deaths per 10,000 infections, respectively, on November 22, 2020.

The study generated other relevant estimates. The diagnosis (detection) rate as of November 22, 2020, that is the proportion of infections that were documented out of all infections that were estimated to have occurred, was assessed at 12.1% (95% CI 12.0–12.1%). The mean duration of acute-care hospitalization was estimated at 8.73 (95% CI 8.62–8.83) days and the mean duration of ICU hospitalization was estimated at 12.30 (95% CI 12.18–12.41) days.

## Discussion

The striking finding of this study is that SARS-CoV-2 morbidity and mortality demonstrated a very strong age dependence. Infection severity, criticality, and fatality increased very rapidly with age, apart for those ≥ 80 years of age possibly because of a survival effect. This was particularly the case for infection criticality and fatality which were limited for those < 50 years of age but increased very rapidly for those ≥ 50 years of age (Figs. 2, 3). This strong age dependence combined with the lower infection exposure in those ≥ 60 years of age (Supplementary Figure S10) and the small proportion of the population ≥ 50 years of age (9%) and ≥ 60 years of age (2%), all contributed together to a low morbidity and mortality in Qatar. Out of every 1000 infections, only 3.7 infections were destined to be severe or critical, and out of every 10,000 infections, only 1.9 infections were destined to end in COVID-19 death (Table 2).

Notably, both SARS-CoV-2 morbidity and mortality in Qatar were not much higher than those typically seen in a seasonal influenza epidemic in the United States^23,24^. This fact, however, needs to be interpreted in context. With the young age structure of the population in Qatar, a seasonal influenza epidemic in this country has a much lower severity than that in the United States. Typically, only a handful of influenza-related deaths are reported every year in Qatar^25^.

Other lines of evidence support these findings. In a survey of ten CMW communities in Qatar, only five severe infections and one critical infection ever occurred in 3233 persons with confirmed infection (antibody and/or PCR positive result), that is an infection severity *and* criticality rate of 2.5 (95% CI 1.1–4.9) per 1000 infections^8^. In another nationwide survey of the CMW population, only seven severe infections and one critical infection ever occurred in 1590 persons with antibody and/or PCR positive result, an infection severity *and* criticality rate of 5.0 (95% CI 2.2–9.9) per 1000 infections^9^. Both of these estimates are in agreement with the present study estimate of 3.7 (95% CI 3.7–3.8) per 1000 infections.

These figures, however, are substantially lower than those estimated elsewhere, often using early epidemic data^26–34^. For instance, the infection acute-care *and* ICU bed hospitalization rate and the infection fatality rate were estimated at 20.4 per 1000 infections and 65.0 per 10,000 infections in the United States, respectively^32^. The fact that the early phases of the epidemic in Europe and the United States heavily affected nursing facilities and care homes of the elderly may have biased many of the early estimates to higher values. It is also possible that our estimates are lower because of the robust accounting of the large pool of undocumented infections in the present study, thanks to the series of serological surveys conducted in Qatar^6–9^. These surveys provided some of the key input data for this modeling study. For instance, the nationwide survey of the CMW population found that only 9.3% (95% CI 7.9–11.0%) of those antibody positive had a prior documented PCR laboratory-confirmed infection^9^. This is in agreement with the diagnosis (detection) rate estimated here at 12.1% (95% CI 12.0–12.1%), as well as growing evidence from other countries indicating that only one in every 10 infections was ever diagnosed during the epidemic’s first wave^26,35–38^. The totality of evidence on the Qatar epidemic also indicates that most infections were asymptomatic or minimally mild to be diagnosed^5–9,39–41^. For instance, a national SARS-CoV-2 PCR survey found that 58.5% of those testing PCR positive reported no symptoms within the preceding two weeks of the survey^6^.

In light of these findings, it is evident that the strong age dependence of SARS-CoV-2 morbidity and mortality is a principal contributor to the low morbidity and mortality seen in Qatar compared to elsewhere. The impact of this strong age dependence is illustrated in Fig. 3B where the age-specific infection fatality rate of Table 2 has been applied to the age structure of other national populations^22^. The infection fatality rate in Italy was found to be tenfold higher than that in Qatar, *only because* of the population’s different age structure. These findings indicate that the infection morbidity and mortality may vary immensely across countries, and will be substantially lower in countries with younger demographic structures, as suggested earlier^12^.

While age appears to be the principal factor, other factors could have also contributed to explaining the low morbidity and mortality in Qatar. Evidence indicates T cell and antibody reactivity against SARS-CoV-2 in unexposed individuals^42–45^, that probably reflects development of T cell and antibody immune memory to circulating ‘common cold’ coronaviruses, which may have in turn led to lower morbidity and mortality^42–45^. The shared-housing dwelling structure in Qatar that contributed to the large SARS-CoV-2 epidemic may have, along with the frequent international travel of Qatar’s expatriate population, also contributed to higher levels of exposure to other common cold coronaviruses^46,47^, thereby inducing high levels of broadly cross-reactive T cell and antibody responses. Such pre-existing immune reactivity^42–45^ may have thus resulted in lower levels of SARS-CoV-2 morbidity and mortality in the population of Qatar.

The resourced healthcare system, which was well below its threshold even at the epidemic peak, may have also contributed to the low observed morbidity and mortality. Emphasis on proactive early hospital admission and treatment, in addition to a cautious approach for SARS-CoV-2 case management, may have limited the number of people who went on to progress to severe or critical disease. This is illustrated in comparing the hospitalization rate in Qatar versus that in the United States, 13.1 versus 20.4^32^ per 1000 infections, and the infection fatality rate, 1.9 versus 65.0^32^ per 10,000 infections, respectively. Though the infection fatality rate was much lower in Qatar, the hospitalization rate was not too different in the two countries. The cautious approach in Qatar that emphasized hospitalization of COVID-19 patients even when this was not indicated per guidelines may have facilitated provision of medical care promptly when COVID-19 cases started to progress to more severe forms of disease.

Limitations may have affected this study. We estimated rates of infection morbidity and mortality, not accounting for potential differences by sex, comorbidities, or other factors. Model estimations are contingent on the validity and generalizability of input data. Available input data were most complete at the national level, and although there could be subpopulation differences in the highly diverse population of Qatar, these could not be factored in the model given insufficient data at the subpopulation level. Despite these limitations, our model, tailored to the complexity of the epidemic in Qatar, was able to reproduce the observed epidemic trends, and provided profound insights about healthcare needs and infection morbidity and mortality.

In conclusion, SARS-CoV-2 morbidity and mortality demonstrate a strikingly strong age dependence. With its young population structure, both morbidity and mortality were low in Qatar, and not much higher than those typically seen in a seasonal influenza epidemic in the United States^23,24^, but they were substantially lower than COVID-19 morbidity and mortality rates in the United States and elsewhere^32–34^. Out of every 1000 infections, only 3.7 infections were severe or critical, and out of every 10,000 infections, only 1.9 infections were fatal. However, these rates would have been much higher if the population of Qatar had a similar demographic structure to that found in Europe or the United States. These findings suggest that SARS-CoV-2 morbidity and mortality may vary immensely by country or region, and that the pandemic expansion in nations with young populations may lead to considerably milder disease burden than currently believed.

## Supplementary information

Supplementary Information.

## Data Availability

All data are available within the manuscript and its supplementary materials.
