# Supplementary material for: SARS-CoV-2 infection hospitalization, severity, criticality, and fatality rates in Qatar
Source: Sci Rep. 2021 Sep 14;11:18182. doi: 10.1038/s41598-021-97606-8 (PMC8440606; doi:10.1038/s41598-021-97606-8)
Supplement: Supplementary file 1 — Supplementary Information. [file 41598_2021_97606_MOESM1_ESM.docx]

**Supporting Information**

**SARS-CoV-2 infection hospitalization, severity, criticality, and fatality rates in Qatar**

Shaheen Seedat, Hiam Chemaitelly, Houssein H. Ayoub, Monia Makhoul, Ghina R. Mumtaz, Zaina Al Kanaani, Abdullatif Al Khal, Einas Al Kuwari, Adeel A. Butt, Peter Coyle, Andrew Jeremijenko, Anvar Hassan Kaleeckal, Ali Nizar Latif, Riyazuddin Mohammad Shaik, Hadi M. Yassine, Mohamed G. Al Kuwari, Hamad Eid Al Romaihi, Mohamed H. Al-Thani, Roberto Bertollini, and Laith J. Abu-Raddad^*^

Address reprints requests or correspondence to Professor Laith J. Abu-Raddad, Infectious Disease Epidemiology Group, World Health Organization Collaborating Centre for Disease Epidemiology Analytics on HIV/AIDS, Sexually Transmitted Infections, and Viral Hepatitis, Weill Cornell Medicine - Qatar, Qatar Foundation - Education City, P.O. Box 24144, Doha, Qatar. Telephone: +(974) 4492-8321. Fax: +(974) 4492-8333. E-mail: [lja2002@qatar-med.cornell.edu](mailto:lja2002@qatar-med.cornell.edu).

**Supplementary Figure S1.** Time course of the SARS-CoV-2 epidemic in Qatar. Daily incidence of (A) documented laboratory-confirmed infections and (B) documented and undocumented infections estimated by the model.

**
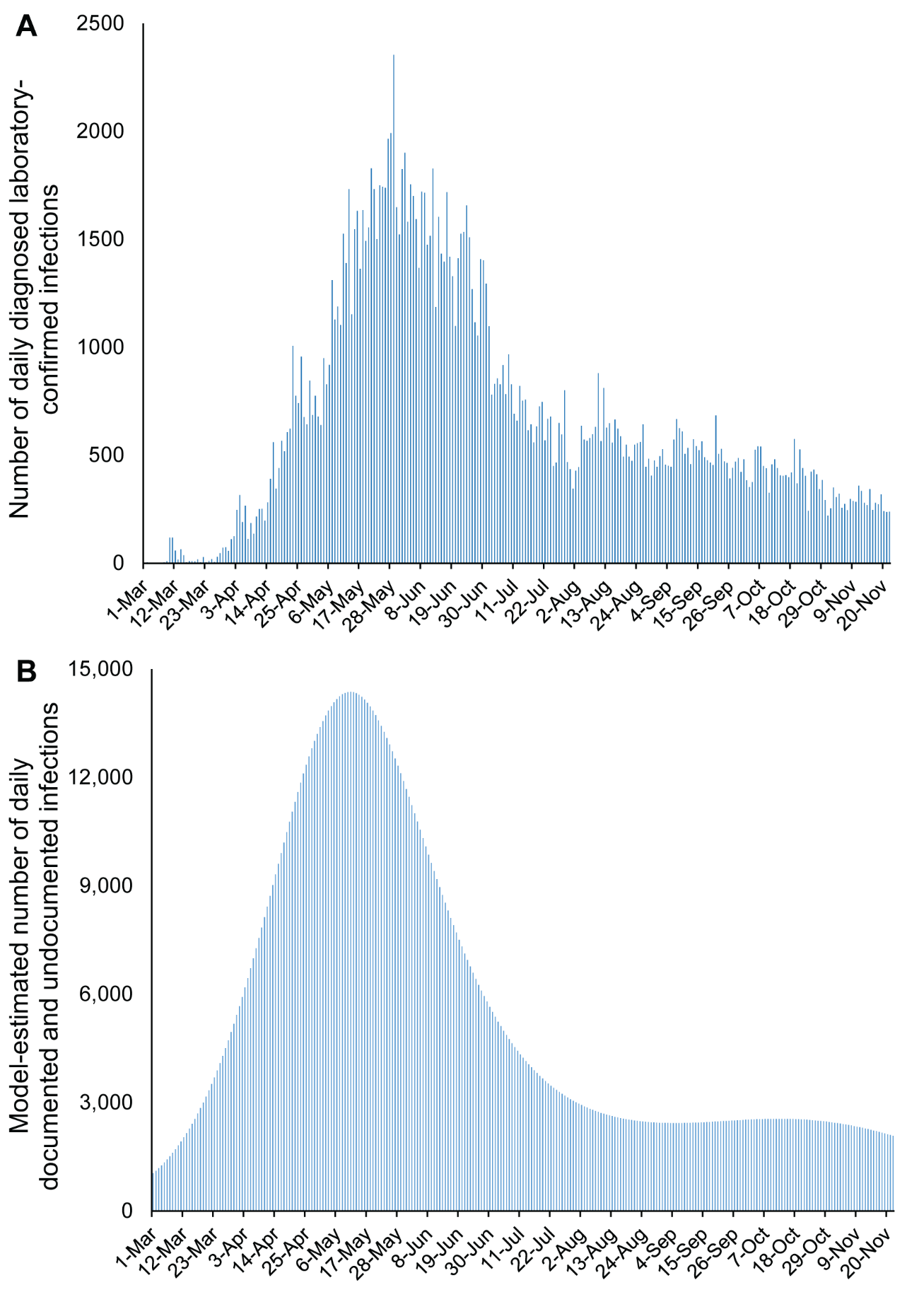
**

**Supplementary Text S1. Mathematical model structure and description**

We constructed an age-structured deterministic mathematical model to describe the [severe acute respiratory syndrome coronavirus 2](https://en.wikipedia.org/wiki/Severe_acute_respiratory_syndrome_coronavirus_2) (SARS-CoV-2) transmission dynamics and disease progression in the population of Qatar (Supplementary Figure S1). The model stratified the population into compartments according to age group (0-9, 10-19, 20-29,…, ≥80 years), infection stage (does not require hospitalization, requires acute-care hospitalization, requires intensive care unit (ICU) hospitalization), and hospitalization stage (acute-care hospitalization, ICU hospitalization). The model also includes five tracking compartments to track infection severity (asymptomatic/moderate/mild infection, severe infection, severe disease, critical infection, critical disease per World Health Organization (WHO) severity classification^1^). The model was structured building on our previously developed models to characterize SARS-CoV-2 epidemics^2-8^.

Epidemic dynamics were described using age-specific sets of coupled nonlinear differential equations. Each age group, $a$, denoted a ten-year age band apart from the last category which grouped together all individuals ≥80 years of age. Qatar’s population size and demographic structure were based on findings of “The Simplified Census of Population, Housing, and Establishments” conducted by Qatar’s Planning and Statistics Authority^9^. Life expectancy was obtained from the United Nations World Population Prospects database^10^.

**Supplementary Figure S2.** Schematic diagram describing the basic structure of the SARS-CoV-2 mathematical model and its associated tracking compartments.


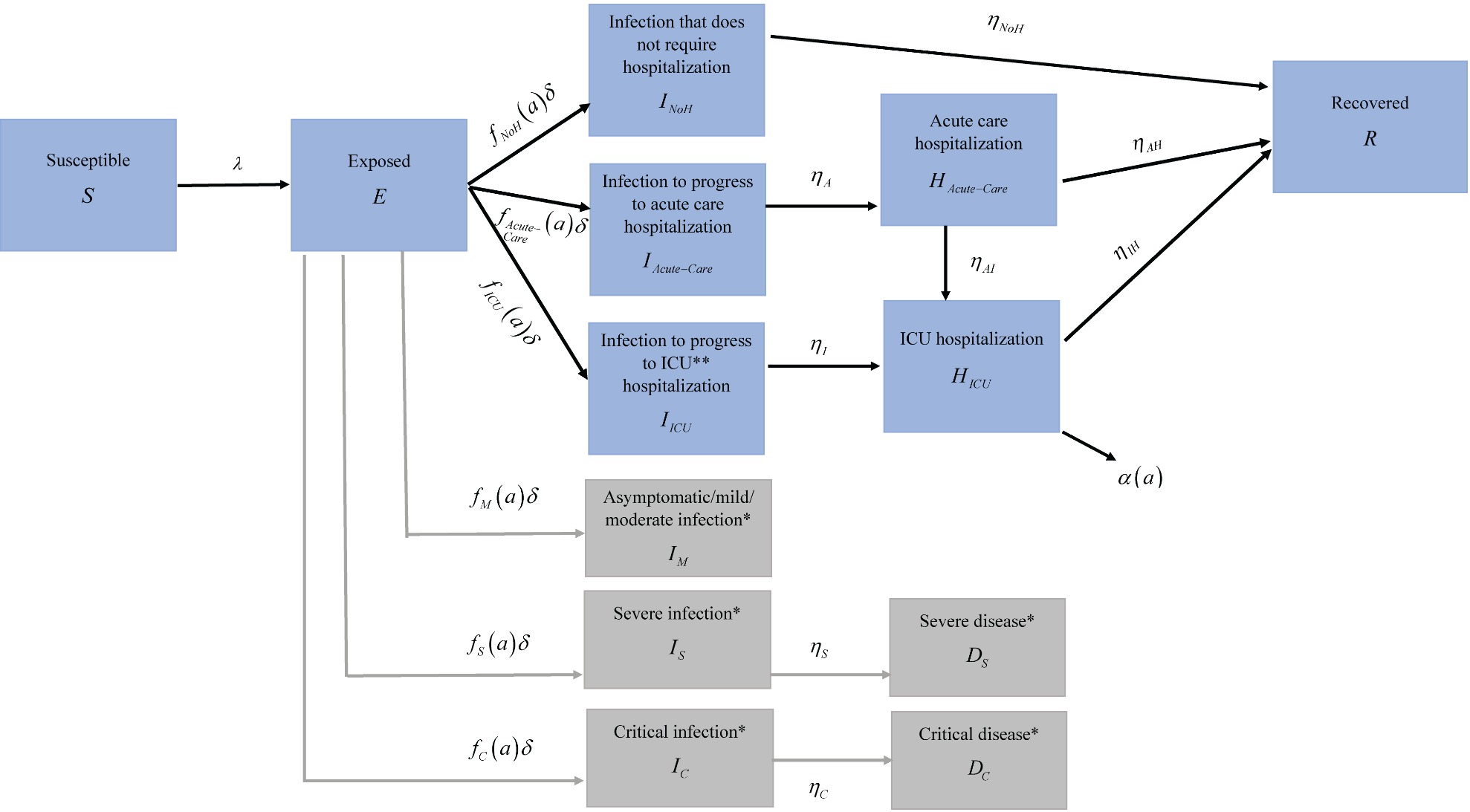


*^*^Per World Health Organization (WHO) infection severity classification^1^*

*^**^ICU: intensive care unit*

The following equations were used to describe the transmission dynamics in the total population:

$$\frac{dS(a)}{dt}=\xi\left(a-1\right)S(a-1)-\left(\lambda\left(a\right)+\mu+\xi\left(a\right)\right)S(a)$$

$$\frac{dE(a)}{dt}=\xi\left(a-1\right)E(a-1)+\lambda\left(a\right)S\left(a\right)-\left(\delta+\mu+\xi\left(a\right)\right)E(a)$$

$$\frac{dI_{NoH}(a)}{dt}=\xi\left(a-1\right)I_{NoH}(a-1)+f_{NoH}\left(a\right)\delta E\left(a\right)-\left(\eta_{NoH}+\mu+\xi\left(a\right)\right)I_{NoH}(a)$$

$$\frac{dI_{Acute-Care}(a)}{dt}=\xi\left(a-1\right)I_{Acute-Care}(a-1)+f_{Acute-Care}(a)\delta E\left(a\right)-\left(\eta_{A}+\mu+\xi\left(a\right)\right)I_{Acute-Care}(a)$$

$$\frac{dI_{ICU}(a)}{dt}=\xi\left(a-1\right)I_{ICU}(a-1)+f_{ICU}(a)\delta E\left(a\right)-\left(\eta_{I}+\mu+\xi\left(a\right)\right)I_{ICU}(a)$$

$$\frac{dH_{Acute-Care}(a)}{dt}=\xi\left(a-1\right)H_{Acute-Care}(a-1)+\eta_{A}I_{Acute-Care}\left(a\right)-\left(\eta_{AH}+\eta_{AI}+\mu+\xi\left(a\right)\right)H_{Acute-Care}(a)$$

$$\frac{dH_{ICU}(a)}{dt}=\xi\left(a-1\right)H_{ICU}(a-1)+\eta_{I}I_{ICU}\left(a\right)+\eta_{AI}H_{Acute-Care}\left(a\right)-\left(\eta_{IH}+\mu+\xi\left(a\right)+\alpha\left(a\right)\right)H_{ICU}(a)$$

$$\frac{dR(a)}{dt}=\xi\left(a-1\right)R(a-1)+\eta_{NoH}I_{NoH}(a)+\eta_{AH}H_{Acute-Care}(a)+\eta_{IH}H_{ICU}(a)-\left(\mu+\xi\left(a\right)\right)R(a)$$

The following equations were used to track the individuals who have asymptomatic/moderate/mild infection, severe infection, severe disease, critical infection, and critical disease per WHO infection severity classification^1^:

$$\frac{dI_{M}(a)}{dt}=\xi\left(a-1\right)I_{M}(a-1)+f_{M}(a)\delta E\left(a\right)-\left(\mu+\xi\left(a\right)\right)I_{M}(a)$$

$$\frac{dI_{S}(a)}{dt}=\xi\left(a-1\right)I_{S}(a-1)+f_{S}(a)\delta E\left(a\right)-\left(\eta_{S}+\mu+\xi\left(a\right)\right)I_{S}(a)$$

$$\frac{dI_{C}(a)}{dt}=\xi\left(a-1\right)I_{C}(a-1)+f_{C}(a)\delta E\left(a\right)-\left(\eta_{C}+\mu+\xi\left(a\right)\right)I_{C}(a)$$

$$\frac{dD_{S}(a)}{dt}=\xi\left(a-1\right)D_{S}(a-1)+\eta_{S}I_{S}\left(a\right)-\left(\mu+\xi\left(a\right)\right)D_{S}(a)$$

$$\frac{dD_{C}(a)}{dt}=\xi\left(a-1\right)D_{C}(a-1)+\eta_{C}I_{C}\left(a\right)-\left(\mu+\xi\left(a\right)\right)D_{C}(a)$$

The definitions of population variables and symbols used in the equations are listed in Supplementary Table S1.

**Supplementary Table S1**. Definitions of population variables and symbols used in the model.

| Symbol | Definition |
| --- | --- |
| $S(a)$ | Susceptible population |
| $E(a)$ | Latently infected population |
| $I_{NoH}\left(a\right)$ | Population that is infectious but with an infection that will not require hospitalization |
| $I_{Acute-Care}\left(a\right)$ | Population that is infectious and that will progress to acute-care hospitalization |
| $I_{ICU}\left(a\right)$ | Population that is infectious and that will progress to ICU hospitalization |
| $H_{Acute-Care}\left(a\right)$ | Population with hospitalization in acute-care beds |
| $H_{ICU}\left(a\right)$ | Population with hospitalization in ICU beds |
| $R\left(a\right)$ | Recovered population |
| $I_{M}\left(a\right)$ | Population with an asymptomatic/moderate/mild infection per WHO infection severity classification^1^ |
| $I_{S}\left(a\right)$ | Population with an infection that will progress to severe disease per WHO infection severity classification^1^ |
| $I_{C}\left(a\right)$ | Population with an infection that will progress to critical disease per WHO infection severity classification^1^ |
| $D_{S}\left(a\right)$ | Population with severe disease per WHO infection severity classification^1^ |
| $D_{C}\left(a\right)$ | Population with critical disease per WHO infection severity classification^1^ |
| $n_{age}$ | Number of age groups |
| $\xi(a)$ | Transition rate from one age group to the next age group. Here $\xi(0)=\xi(n_{age})=0$ |
| $\sigma\left(a\right)$ | Susceptibility profile to the infection in each age group |
| $1/\delta$ | Duration of latent infection before onset of infectiousness |
| $\beta$ | Average rate of infectious contacts |
| $1/\eta_{NoH}=1/\eta_{A}=1/\eta_{I}$ | Duration of infectiousness |
| $1/\eta_{AH}$ | Duration of acute-care hospitalization following hospital admission and prior to recovery |
| $1/\eta_{IH}$ | Duration of ICU hospitalization following hospital admission and prior to recovery |
| $1/\eta_{AI}$ | Duration of acute-care hospitalization prior to admission to ICU for those infected individuals that are transferred from acute-care to ICU |
| $1/\eta_{S}$ | Duration of severe infection prior to onset of severe disease |
| $1/\eta_{C}$ | Duration of critical infection prior to onset of critical disease |
| $\mu$ | Natural death rate |
| $\alpha\left(a\right)$ | Disease mortality rate in each age group |
| $f_{NoH}(a)$ | Proportion of infections that will progress to be infections that will not require hospitalization |
| $f_{Acute-Care}(a)$ | Proportion of infections that will progress to be infections that require hospitalization in acute-care beds |
| $f_{ICU}(a)$ | Proportion of infections that will progress to be infections that require hospitalization in ICU beds |
| $f_{M}(a)$ | Proportion of infections that are asymptomatic/moderate/mild per WHO infection severity classification^1^ |
| $f_{S}(a)$ | Proportion of infections that will progress to be severe infections per WHO infection severity classification^1^ |
| $f_{C}(a)$ | Proportion of infections that will progress to be critical infections per WHO infection severity classification^1^ |

The force of infection (hazard rate of infection) experienced by each susceptible population, $S(a)$, is given by

$$\lambda\left(a\right)=\beta\left(t\right)\sigma\left(a\right)\sum_{a'=1}^{n_{age}} H_{_{a,a'}}\frac{I_{NoH}(a')+I_{Acute-Care}(a')+I_{ICU}(a')}{\left[S(a')+E(a')+I_{NoH}(a')+I_{Acute-Care}(a')+I_{ICU}(a')+H_{Acute-Care}(a')+H_{ICU}(a')+R(a')\right]}$$

Here, $\beta\left(t\right)$ is the time-dependent average rate of infectious contacts and $\sigma\left(a\right)$ is the susceptibility profile to the infection in each age group $a$. To account for temporal variation in the basic reproduction number, we incorporated a temporal variation in $\beta$ that was parameterized through a combination of Woods-Saxon and Logistic functions.

This function was mathematically designed to describe and characterize the time evolution of the level of risk of exposure before and after easing of restrictions. It was informed by our knowledge of SARS-CoV-2 epidemiology in Qatar^11^, and it provided a robust fit to the data. Here $a_{1}$, $a_{2}$, $b_{1}$, $b_{2}$, $c_{1}$, and $c_{2}$ are fitting parameters.

The probability that an individual in the $a$ age group will mix with an individual in the $a'$ age group is determined by an age-mixing matrix, $H_{_{a,a'}}$, given by

$$H_{_{a,a'}}=e_{Age}\delta_{a,a'}+(1-e_{Age})\frac{S(a')+E(a')+I_{NoH}(a')+I_{Acute-Care}(a')+I_{ICU}(a')+H_{Acute-Care}(a')+H_{ICU}(a')+R(a')}{\sum_{a''=1}^{n_{age}} \left[S(a'')+E(a'')+I_{NoH}(a'')+I_{Acute-Care}(a'')+I_{ICU}(a'')+H_{Acute-Care}(a'')+H_{ICU}(a'')+R(a'')\right]}$$

Here, $\delta_{a,a'}$ is the identity matrix and $e_{Age}\in\left[0,1\right]$ measures the degree of assortativeness in the age mixing. At the extreme $e_{Age}=0$, the mixing is fully proportional, while at the other extreme, $e_{Age}=1$, the mixing is fully assortative, that is individuals mix only with members in their own age group.

**Supplementary Text S2.** Parameter values, data input, and model fitting

Model input parameters were based on best available empirical data for SARS-CoV-2 natural history and epidemiology. Model input parameter values and supporting evidence are listed in Supplementary Table S2.

**Supplementary Table S2.** Model input parameters.

| **Parameter** | **Symbol** | **Value** | **Justification** |
| --- | --- | --- | --- |
| Duration of latent infection | $1/\delta$ | 3.69 days | Based on existing estimate^12^ and based on a median incubation period of 5.1 days^13^ adjusted by observed viral load among infected persons^14^ and reported transmission before onset of symptoms^15^ |
| Duration of infectiousness | $1/\eta_{NoH}$;$1/\eta_{A}$; $1/\eta_{I}$ | 3.48 days | Based on existing estimate^12^ and based on observed time to recovery among persons with mild infection^12,16^ and observed viral load in infected persons^14,15,17^ |
| Life expectancy in Qatar |  | 80.7 years | United Nations World Population Prospects database^10^ |

The model was fitted to extensive sources of data thanks to the centralized and standardized databases of SARS-CoV-2 testing, infection, COVID-19 disease, hospitalization, and severity as well as to findings of ongoing epidemiologic studies in Qatar. Data included: 1) time-series of number of PCR laboratory-confirmed SARS-CoV-2 infections, 2) distribution of PCR laboratory-confirmed infections by age group, 3) time-series of SARS-CoV-2 PCR testing positivity rate, 4) fraction of PCR laboratory-confirmed SARS-CoV-2 infected persons aged >60 years old, 5) a series of PCR and serological surveys, 6) age-distribution of SARS-CoV-2 antibody positivity, 7) time-series of new/daily hospital admissions in acute-care beds and in ICU beds, 8) age distribution of hospital admissions in acute-care beds and in ICU beds, 9) fraction of individuals admitted to ICU beds from acute-care beds, 10) time-series of current hospital occupancy in acute-care beds and in ICU beds, 11) time-series of new/daily severe infections and critical infections per WHO infection severity classification^1^, 12) age distribution of severe and critical infections per WHO infection severity classification^1^, 13) time series of COVID-19 deaths, and 14) age distribution of COVID-19 deaths.

A Bayesian method, based on incremental mixture importance sampling with shotgun optimization^18,19^, was used to conduct the model fitting and generate posteriors and credible sets. In the first stage (initialization), the importance sampling distribution was initialized by drawing a large number of points from the prior distribution and weights were attached to each point using their respective log-likelihood.

In the second stage (shot-gun optimization), multiple sequential optimizations were carried out. In each of these, the point of the importance sampling distribution with the maximum weight was computed, and a new sample set was drawn from a Gaussian distribution centered at this point. The local maximum of this sample set (point with largest log-likelihood) was then identified. The importance sampling distribution was then updated to exclude the points that are farthest from this local maximum in terms of Mahalanobis distance. A new sample set was then drawn from a Gaussian distribution centered at the current local maximum and was added to the importance sampling distribution. At the end of the shot-gun optimization stage, a candidate importance sampling distribution was obtained.

In the third stage (importance sampling), weights were attached to each point in the importance sampling distribution and sampling was iterated until convergence in the distribution of the importance sampling distribution. In the fourth stage (resampling), points were resampled from the importance sampling distribution to construct the posterior distribution of each estimated parameter.

**Supplementary Table S3.** Goodness-of-fit in terms of likelihood of the model fitting each dataset.

| **Empirical dataset** | **Model fitting likelihood** |
| --- | --- |
| Time-series of number of PCR laboratory-confirmed SARS-CoV-2 infections | 80.52% |
| Distribution of PCR laboratory-confirmed infections by age group | 82.34% |
| Time-series of SARS-CoV-2 PCR testing positivity rate | 81.81% |
| Fraction of PCR laboratory-confirmed SARS-CoV-2 infections aged >60 years | 98.10% |
| Series of PCR surveys | 89.31% |
| Age-distribution of SARS-CoV-2 antibody positivity | 99.17% |
| Time-series of new/daily hospital admissions in acute-care and in ICU beds | 98.87% |
| Age distribution of hospital admissions in acute-care and in ICU beds | 99.95% |
| Fraction of ICU admissions that are being transferred from acute-care beds | 99.99% |
| Time-series of current hospital occupancy in acute-care and in ICU beds | 93.53% |
| Time-series of new/daily severe infections and critical infections^*^ | 99.43% |
| Age distribution of severe and critical infections^*^ | 99.96% |
| Age distribution of COVID-19 deaths | 99.98% |

^*^Per World Health Organization (WHO) infection severity classification^1^

**Supplementary Figure S3.** Posterior distribution of the age-specific infection acute-care bed hospitalization rate.

**
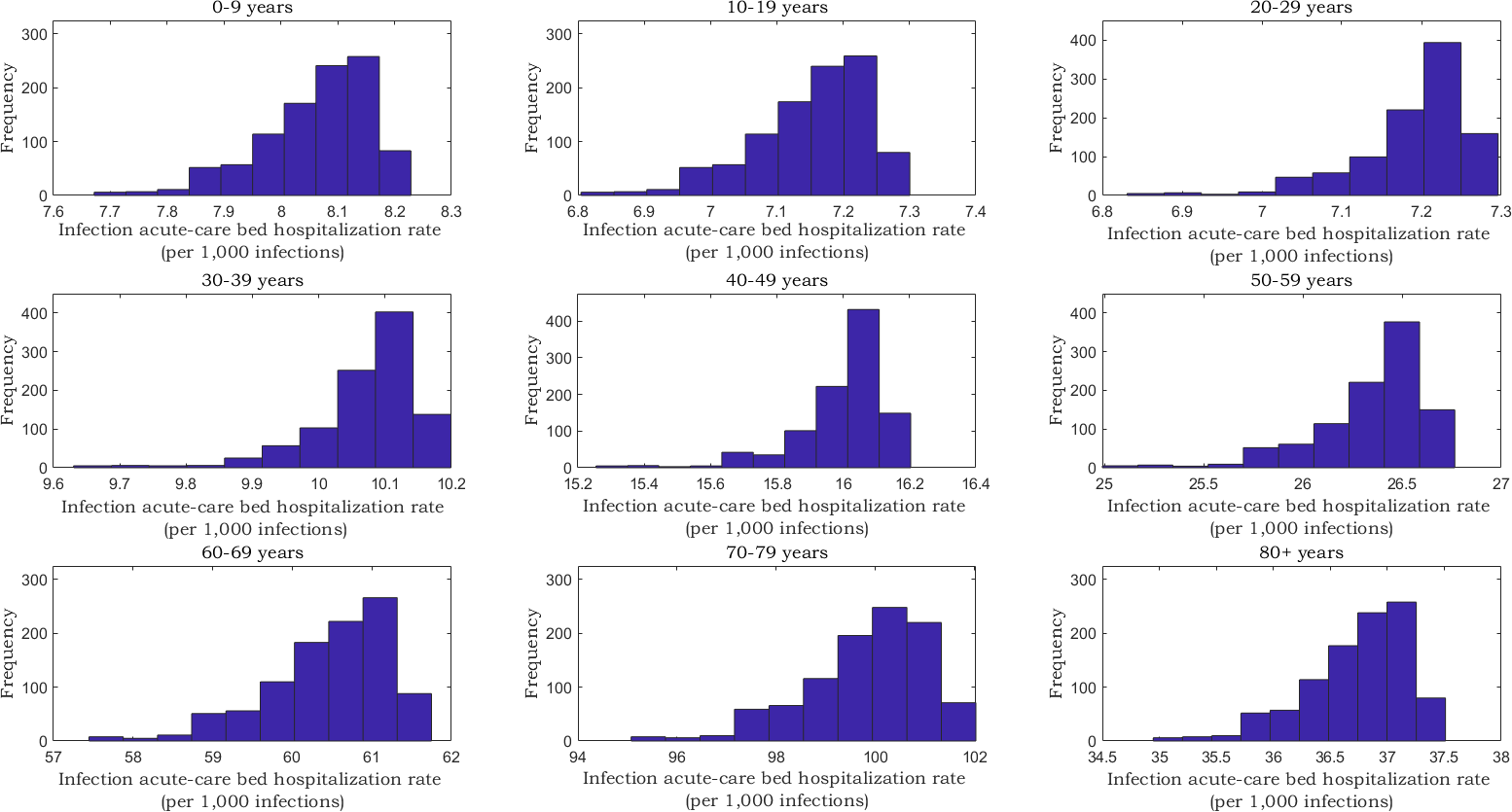
**

**Supplementary Figure S4.** Posterior distribution of the age-specific infection ICU bed hospitalization rate.


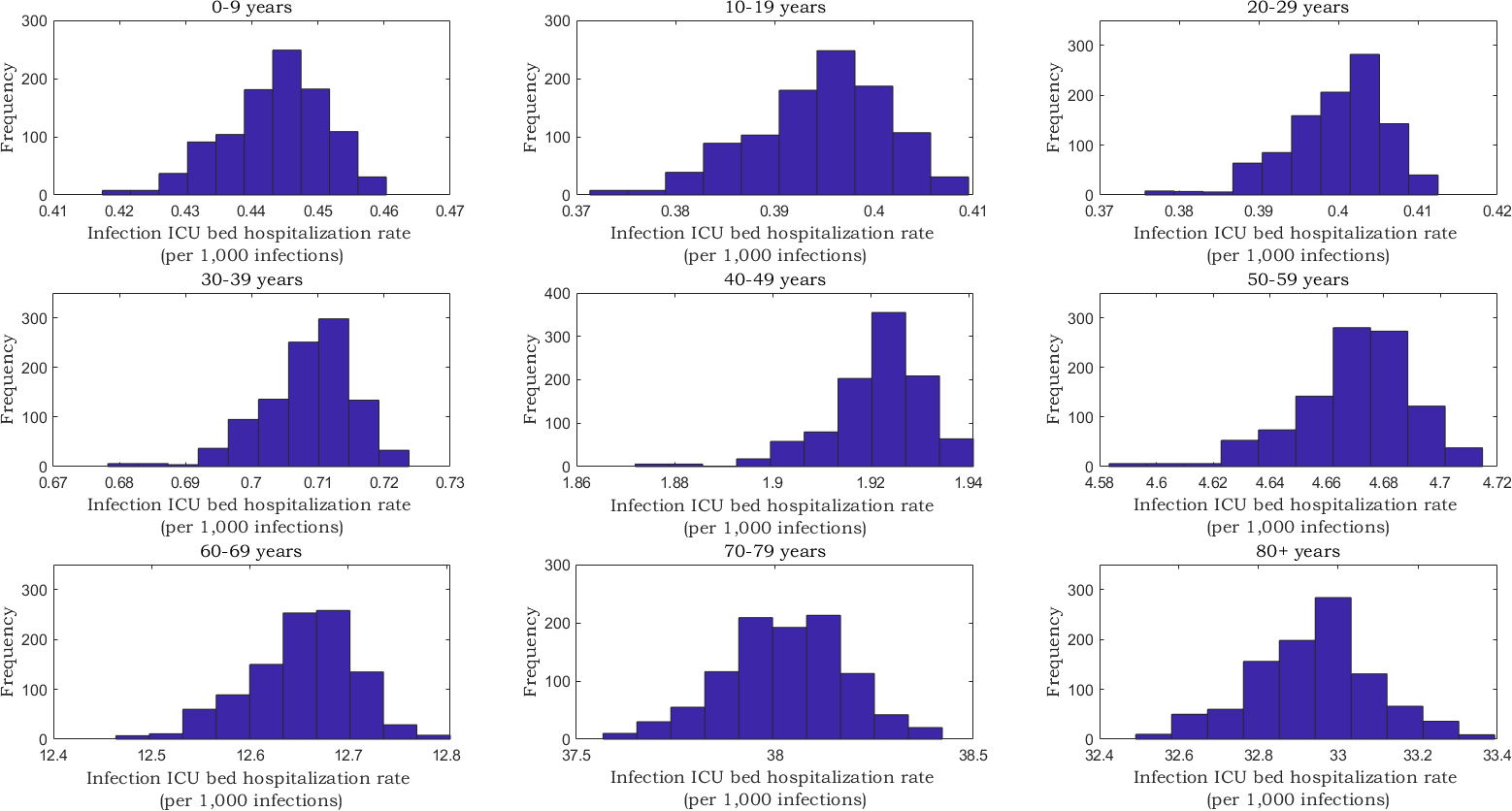


**Supplementary Figure S5.** Posterior distribution of the age-specific infection severity rate. Classification of infection severity was per WHO severity classification^1^.

**
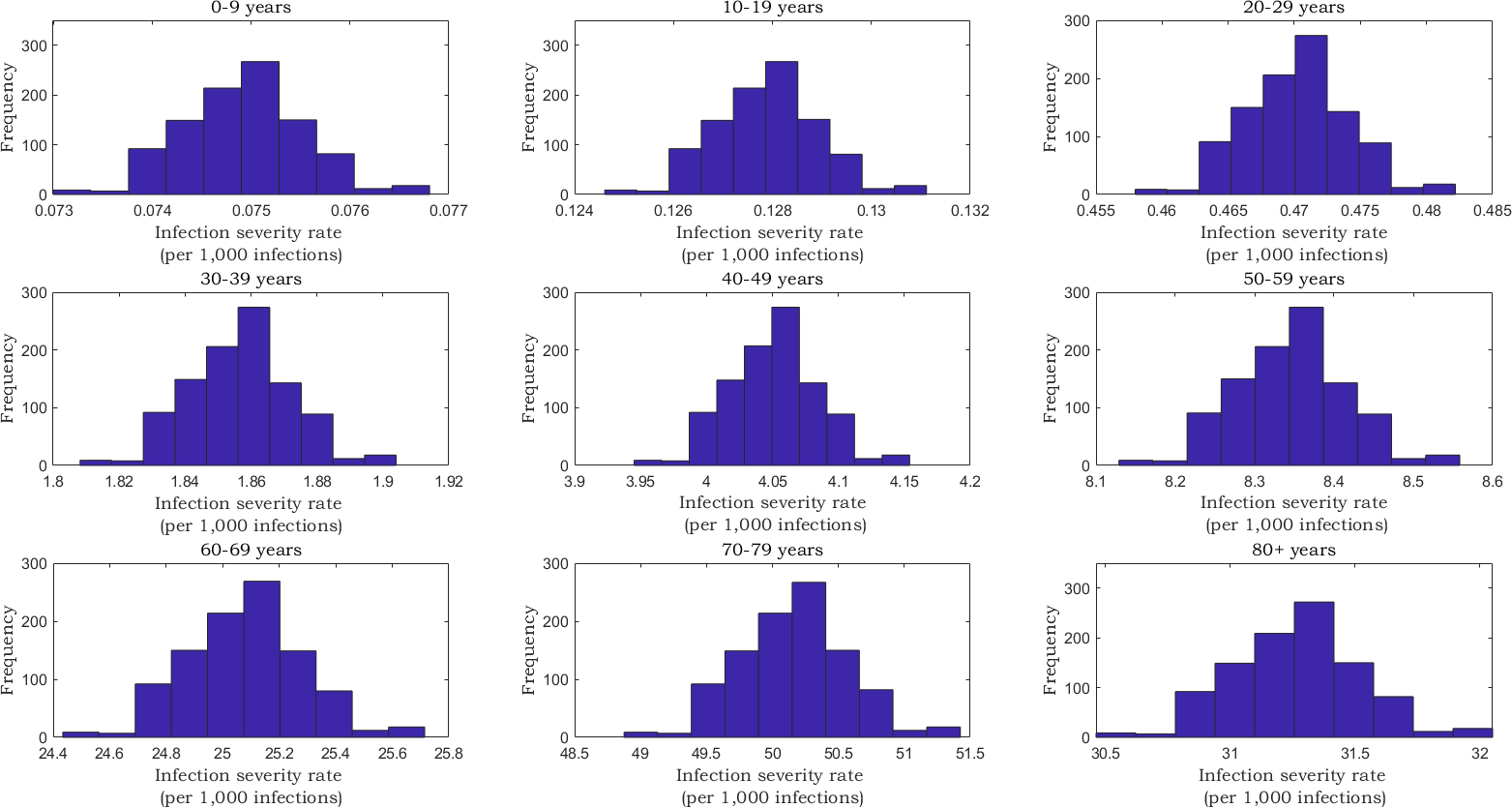
**

**Supplementary Figure S6.** Posterior distribution of the age-specific infection criticality rate. Classification of infection criticality was per WHO severity classification^1^.

**
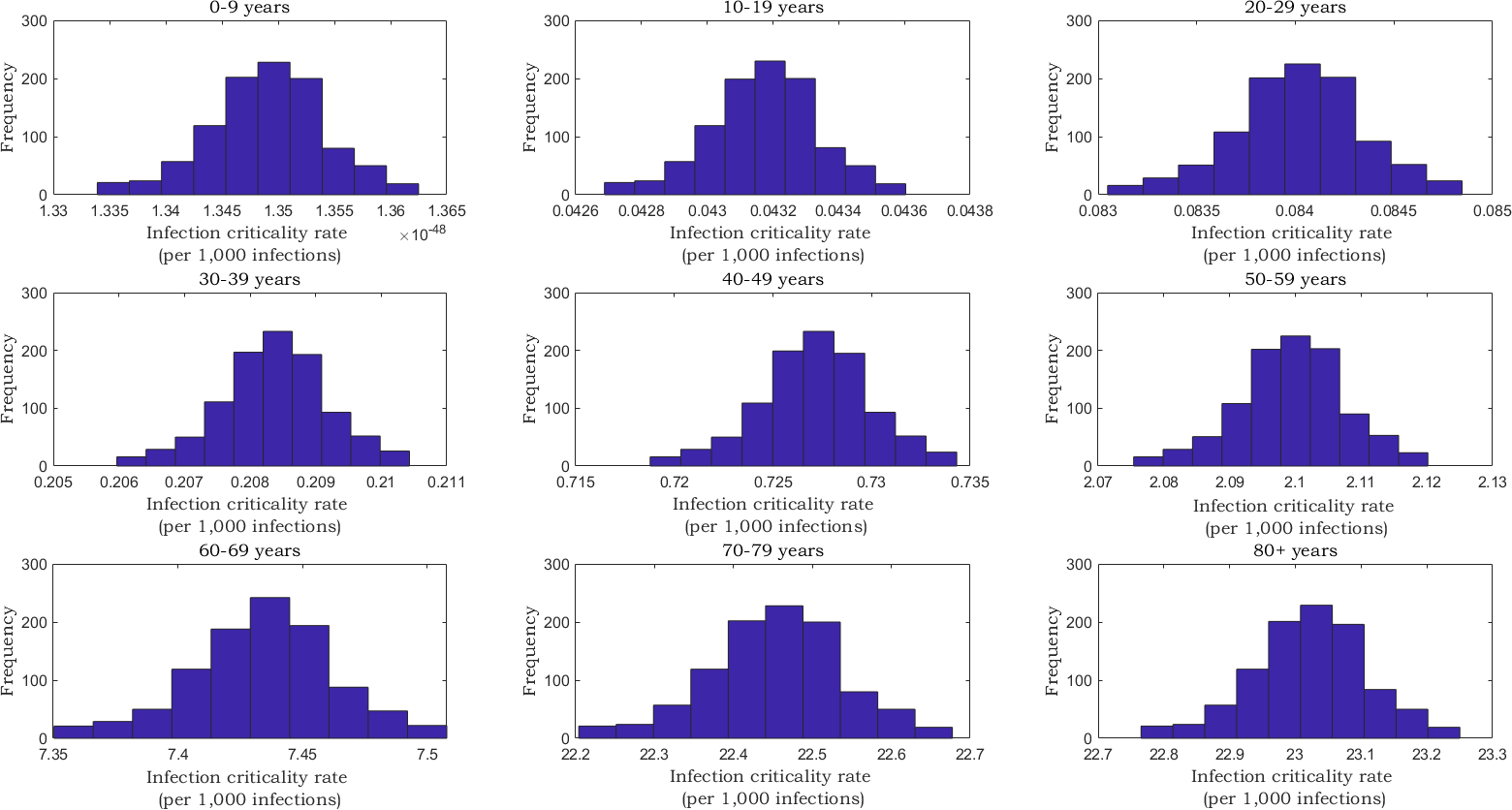
**

**Supplementary Figure S7.** Posterior distribution of the age-specific infection fatality rate.

**
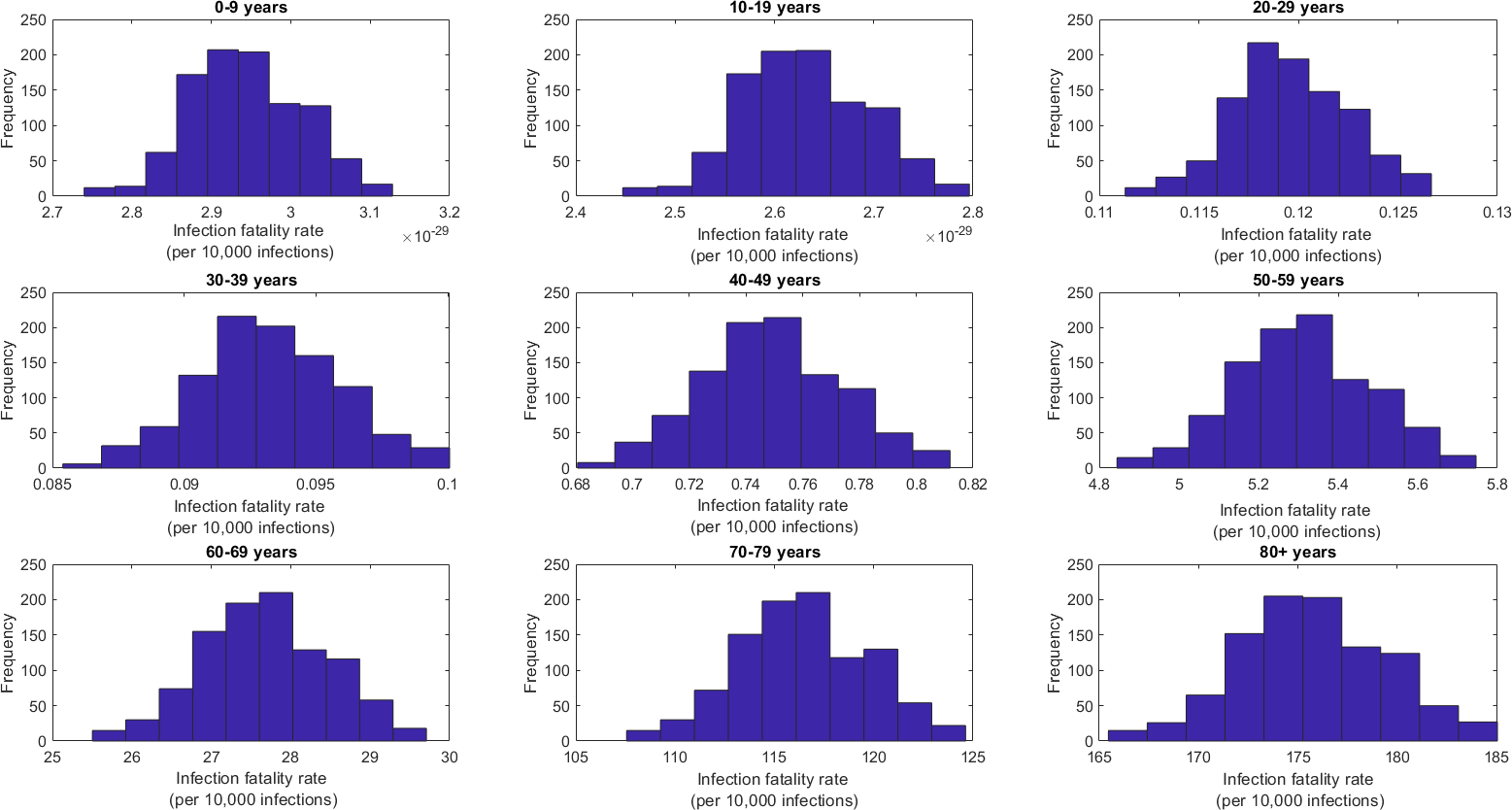
**

**Supplementary Figure S8.** Posterior distribution of the overall (total population of all age groups) A) infection acute-care bed hospitalization rate, B) infection ICU bed hospitalization rate, C) infection severity rate, and D) infection criticality rate.

**
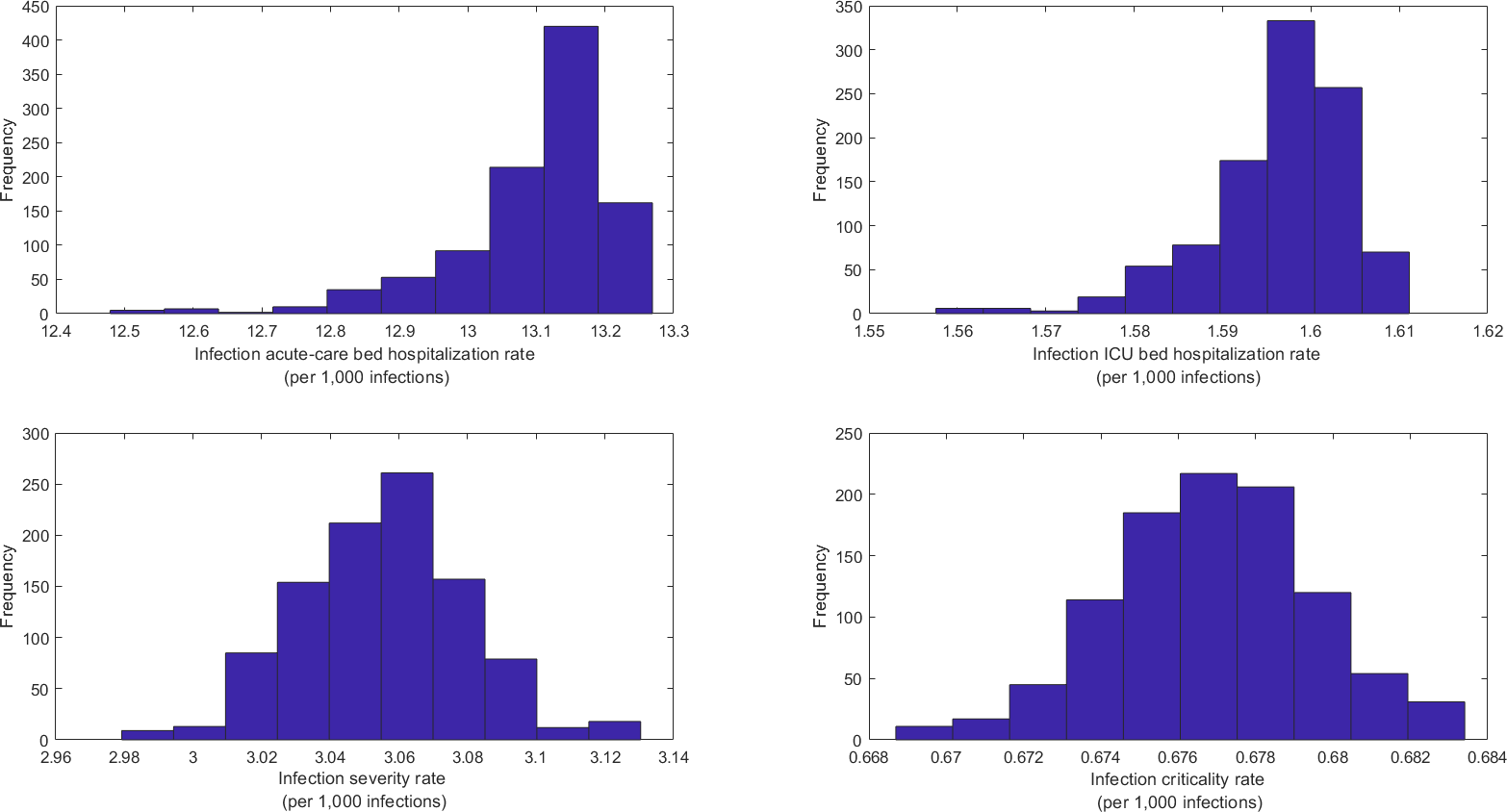
**

**Supplementary Figure S9.** Posterior distribution of the overall (total population of all age groups) infection fatality rate.

**
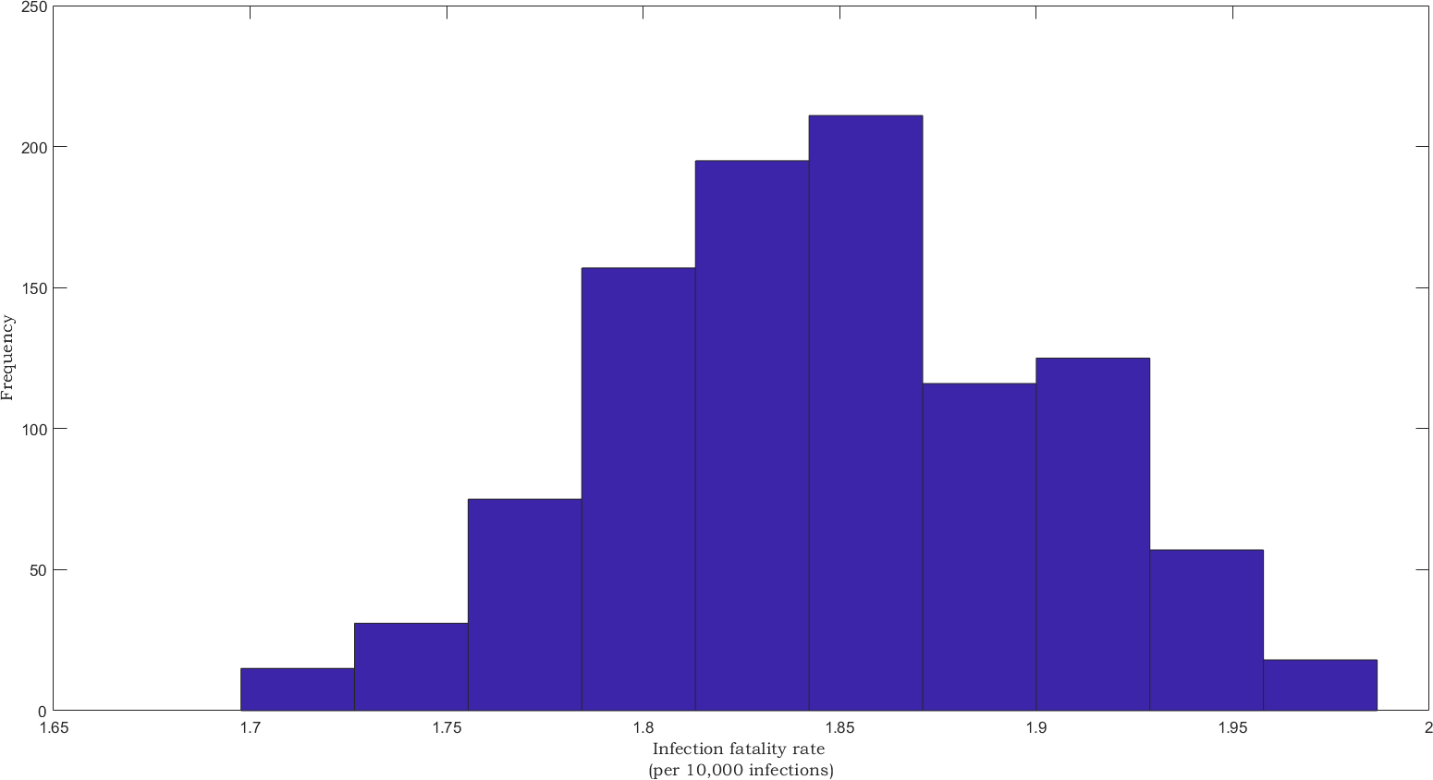
**

**Supplementary Figure S10.** The SARS-CoV-2 age-specific antibody prevalence (seroprevalence) in the total population of Qatar based on compilation and analysis of seroprevalence data from a series of serological studies^11,20-22^.

**
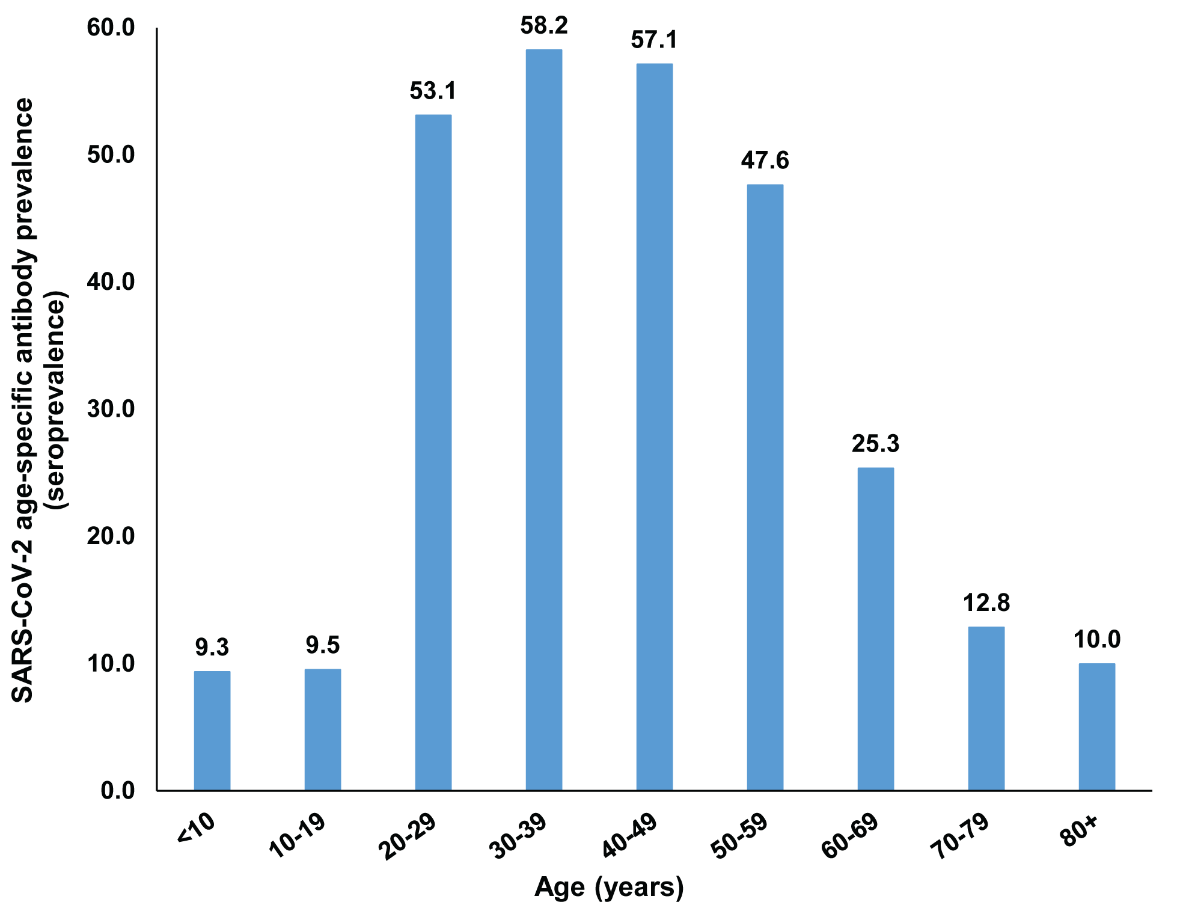
**

**Supplementary References**

1 World Health Organization. Clinical management of COVID-19. Available from: <https://www.who.int/publications-detail/clinical-management-of-covid-19>. Accessed on: May 31st 2020. (2020).

2 Ayoub, H. H. *et al.* Age could be driving variable SARS-CoV-2 epidemic trajectories worldwide. *Plos One* **15**, doi:10.1371/journal.pone.0237959 (2020).

3 Ayoub, H. H. *et al.* Characterizing key attributes of the epidemiology of COVID-19 in China: Model-based estimations. *Global Epidemiology* **100042**, doi:<https://doi.org/10.1016/j.gloepi.2020.100042> (2020).

4 Makhoul M. *et al.* Epidemiological impact of SARS-CoV-2 vaccination: Mathematical modeling analyses. *Vaccines* **8**, doi:10.3390/vaccines8040668 (2020).

5 Makhoul, M. *et al.* Analyzing inherent biases in SARS-CoV-2 PCR and serological epidemiologic metrics. *medRxiv*, 2020.2008.2030.20184705, doi:10.1101/2020.08.30.20184705 (2020).

6 Ayoub, H. H. *et al.* Mathematical modeling of the SARS-CoV-2 epidemic in Qatar and its impact on the national response to COVID-19. *J Glob Health* **11**, 05005 (2021).

7 Makhoul, M., Chemaitelly, H., Ayoub, H. H., Seedat, S. & Abu-Raddad, L. J. Epidemiological Differences in the Impact of COVID-19 Vaccination in the United States and China. *Vaccines (Basel)* **9**, doi:10.3390/vaccines9030223 (2021).

8 Abu-Raddad, L. J. *et al.* Characterizing the Qatar advanced-phase SARS-CoV-2 epidemic. *medRxiv*, 2020.2007.2016.20155317v20155312 (non-peer-reviewed preprint), doi:10.1101/2020.07.16.20155317 (2020).

9 Planning and Statistics Authority-State of Qatar. The Simplified Census of Population, Housing & Establishments. Available from: <https://www.psa.gov.qa/en/statistics/Statistical%20Releases/Population/Population/2018/Population_social_1_2018_AE.pdf> Accessed on: April 2, 2020. (2019).

10 United Nations Department of Economic and Social Affairs Population Dynamics. The 2019 Revision of World Population Prospects. Available from <https://population.un.org/wpp/>. Accessed on March 1st, 2020. (2020).

11 Abu-Raddad, L. J. *et al.* Characterizing the Qatar advanced-phase SARS-CoV-2 epidemic. *Scientific Reports* **11**, 6233, doi:10.1038/s41598-021-85428-7 (2021).

12 Li, R. *et al.* Substantial undocumented infection facilitates the rapid dissemination of novel coronavirus (SARS-CoV2). *Science* **368**, 489-493, doi:10.1126/science.abb3221 (2020).

13 Lauer, S. A. *et al.* The Incubation Period of Coronavirus Disease 2019 (COVID-19) From Publicly Reported Confirmed Cases: Estimation and Application. *Ann Intern Med* **172**, 577-582, doi:10.7326/M20-0504 (2020).

14 Zou, L. *et al.* SARS-CoV-2 Viral Load in Upper Respiratory Specimens of Infected Patients. *N Engl J Med* **382**, 1177-1179, doi:10.1056/NEJMc2001737 (2020).

15 Rothe, C. *et al.* Transmission of 2019-nCoV Infection from an Asymptomatic Contact in Germany. *N Engl J Med* **382**, 970-971, doi:10.1056/NEJMc2001468 (2020).

16 World Health Organization. Report of the WHO-China Joint Mission on Coronavirus Disease 2019 (COVID-19). Available from :<https://www.who.int/docs/default-source/coronaviruse/who-china-joint-mission-on-covid-19-final-report.pdf>. Accessed on March 10, 2020. (2020).

17 He, X. *et al.* Temporal dynamics in viral shedding and transmissibility of COVID-19. *Nat Med* **26**, 672-675, doi:10.1038/s41591-020-0869-5 (2020).

18 Raftery, A. E. & Bao, L. Estimating and projecting trends in HIV/AIDS generalized epidemics using incremental mixture importance sampling. *Biometrics* **66**, 1162-1173 (2010).

19 Jonoska Stojkova, B. & Campbell, D. A. Incremental Mixture Importance Sampling With Shotgun Optimization. *Journal of Computational and Graphical Statistics* **28**, 806-820 (2019).

20 Coyle, P. V. *et al.* SARS-CoV-2 seroprevalence in the urban population of Qatar: An analysis of antibody testing on a sample of 112,941 individuals. *iScience*, 102646, doi:10.1016/j.isci.2021.102646 (2021).

21 Jeremijenko, A. *et al.* Herd Immunity against Severe Acute Respiratory Syndrome Coronavirus 2 Infection in 10 Communities, Qatar. *Emerg Infect Dis* **27**, 1343-1352, doi:10.3201/eid2705.204365 (2021).

22 Al-Thani, M. H. *et al.* SARS-CoV-2 infection is at herd immunity in the majority segment of the population of Qatar. *Open Forum Infectious Diseases*, doi:10.1093/ofid/ofab221 (2021).
